# Isorhamnetin Inhibits Human Gallbladder Cancer Cell Proliferation and Metastasis via PI3K/AKT Signaling Pathway Inactivation

**DOI:** 10.3389/fphar.2021.628621

**Published:** 2021-02-17

**Authors:** Tianyu Zhai, Xiaoyu Zhang, Zhenyu Hei, Longyang Jin, Chao Han, Audrey Tsznam Ko, Xiaofeng Yu, Jiandong Wang

**Affiliations:** ^1^ Department of General Surgery, Xinhua Hospital, Affiliated to Shanghai Jiao Tong University School of Medicine, Shanghai, China; ^2^ Shanghai Research Center of Biliary Tract Disease, Shanghai, China; ^3^ Shanghai Key Laboratory of Biliary Tract Disease Research, Shanghai, China; ^4^ Department of Colorectal Surgery, The Sixth Affiliated Hospital of Sun Yat-sen University, Guangzhou, China; ^5^ Department of General Surgery, Shanghai General Hospital, Shanghai Jiao Tong University School of Medicine, Shanghai, China; ^6^ Faculty of Medicine, Imperial College London, London, United Kingdom; ^7^ Department of General Surgery, People's Hospital of Gaoxin District, Suzhou, China

**Keywords:** isorhamnetin, gallbladder cancer, tumor progression, PI3K/akt pathway, apoptosis

## Abstract

Gallbladder cancer (GBC) is the most common biliary tract tumor with a poor prognosis. Isorhamnetin is a flavonoid compound extracted from *Hippophae rhamnoides L.* and has several pharmacological effects including anti-inflammatory and anti-cancer properties. We treated GBC-SD and NOZ of GBC cell lines with different isorhamnetin concentrations *in vitro*. A cell counting kit-8 (CCK-8) assay, transwell assay, Hoechst 33342 stain assay, flow cytometric analysis, and a colony-forming assay were performed to investigate the effect of isorhamnetin on the proliferation, apoptosis, metastasis, and cycle arrest of GBC cells. A western blotting assay was conducted to explore the related protein expression level of GBC cells. A mice xenograft model and immunohistochemistry staining were employed to assess the effect of isorhamnetin *in vivo*. Isorhamnetin was found to suppress cell proliferation and metastasis, and trigger apoptosis and arrest the G2/M phase in GBC cells via the inactivation of the PI3K/AKT signaling cascade. Our findings are of clinical significance in providing a novel treatment approach for GBC.

## Introduction

Gallbladder cancer (GBC) is the most common biliary tract malignancy (Li et al., 2014). GBC often progresses gradually with a late diagnosis because of its nonspecific symptoms (Hundal and Shaffer, 2014). Therefore, most GBC patients are difficult to cure since radical surgery is the only efficient treatment (Misra et al., 2003). The overall prognosis of GBC is very poor and the survival rate of GBC patients after five years is only 5% (Wolpin and Mayer, 2010; Shu et al., 2017; Zhai et al., 2017). Therefore, novel potential biomarkers and treating strategies for GBC patients need to be urgently identified.

**FIGURE 1:**
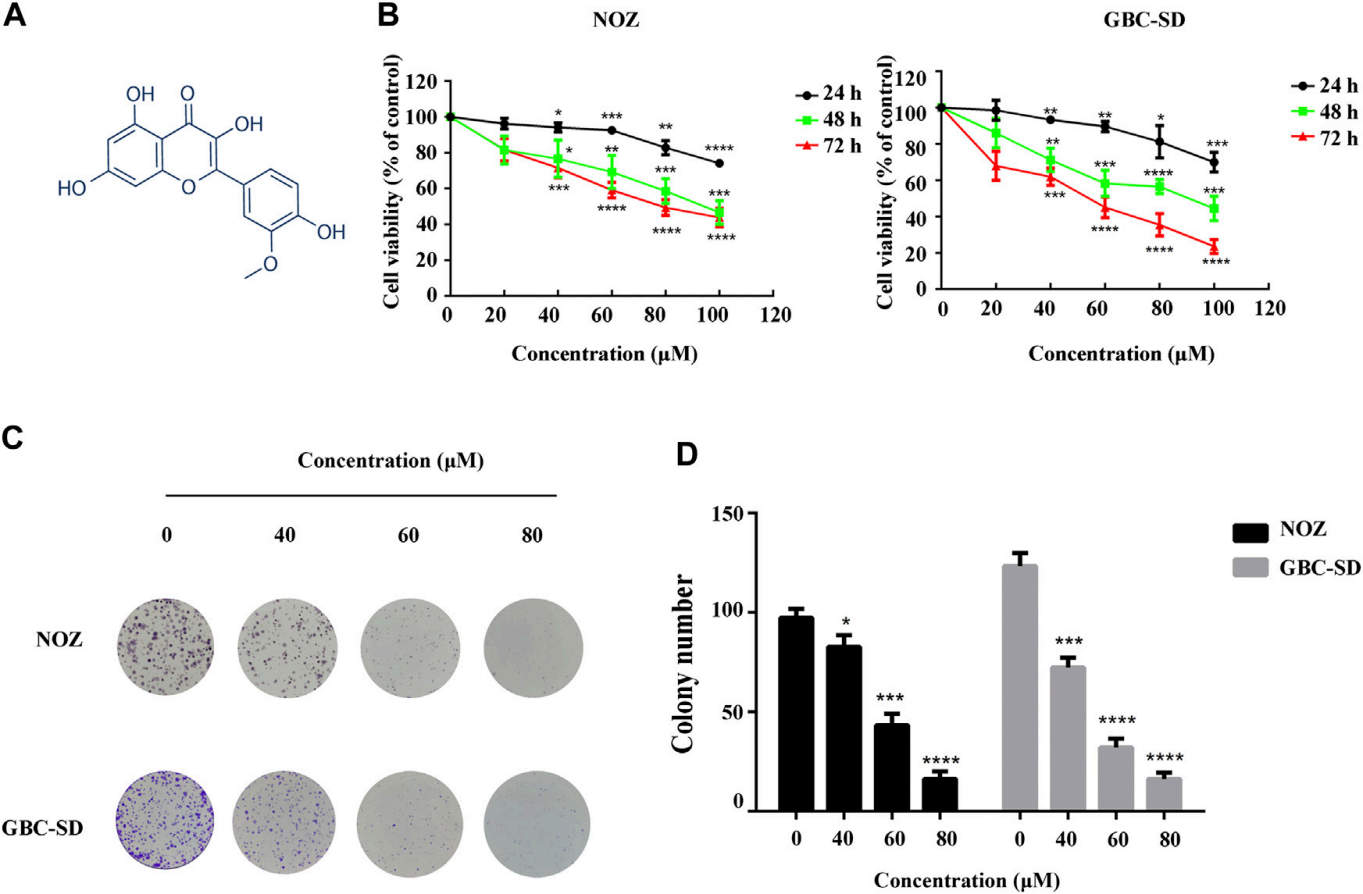
Isorhamnetin suppresses GBC cell proliferation *in vitro*. **(A)** Chemical structure of isorhamnetin. **(B)** The cell viability of GBC cells assessed using CCK-8 assay after isorhamnetin treatment (0, 40, 60, 80, and 100 μM) for 24, 48, and 72 h. **(C)** Colony formation assay of NOZ and GBC-SD cells exposed to isorhamnetin (0, 40, 60, , and 80 μM) for 10 days. **(D)** The colony numbers (>100 cells/colony) were calculated manually. *p < 0.05, ***p < 0.001, ****p < 0.0001 relative to the control group.

Isorhamnetin (Figure 1A) is a flavonoid compound extracted from the leaves, flowers, and fruits of *Hippophae rhamnoides L.* (Teng et al., 2006), Ginkgo biloba L., and other plants (Thota et al., 2018). Studies have indicated that isorhamnetin has diverse pharmacological effects on cardiovascular diseases (Huang et al., 2016), rheumatism (Gupta et al., 2010), and hemorrhage. It also contains pharmacodynamics against hyperuricemia (Adachi et al., 2019) and pulmonary fibrosis (Zheng et al., 2019). Isorhamnetin can influence various types of tumors including lung, gastric, skin, colorectal, and esophageal (Ma et al., 2007; Kim et al., 2011; Ramachandran et al., 2012; Saud et al., 2013; Ruan et al., 2015). Furthermore, several carcinogenic signaling cascades, such as NF-κB, PI3K/AKT, and MAPK cascades, are involved in the pharmacological effects of isorhamnetin on GBC cells. The PI3K/AKT signaling cascade plays essential roles in various aspects of cell growth and survival during tumorigenesis, including proliferation, apoptosis, and immunity (Arthur and Ley, 2013; Peti and Page, 2013; Porta et al., 2014).

In this study, we demonstrate that isorhamnetin can effectively suppress the proliferation, cellular morphology, and metastasis of GBC cells, both *in vivo* and *in vitro* via inactivating the PI3K/AKT signaling cascade, which may provide a promising treatment agent for GBC patients.

## Materials and Methods

### Chemical Preparation and Cell Culture

Isorhamnetin (Iso, purity ≥98%, Cat number: B21554) was sourced from the Shanghai Yuanye Bio-Technology Co., Ltd. and dissolved in DMSO, SC79 (Cat number: abs810216) was sourced from the Shanghai Univ-bio Bio-Technology Co., Ltd. and dissolved in DMSO. NOZ, and the GBC-SD of human GBC cell lines were sourced from the Cell Bank of the Chinese Academy of Sciences (Shanghai, China), and the STR analysis can be found in Supplementary Material. These cells were cultured in William’s medium (Gibco, Grand Island, NY, United States), and DMEM (Gibco, Grand Island, NY, United States). We cultured the cells in a medium with fetal bovine serum (10%) (FBS, Gibco) and ampicillin/streptomycin (1%) in an incubator with 5% CO_2_ at 37°C.

### Cell Proliferation Assay

The cell counting kit-8 (CCK-8) was used to perform cell viability analysis. The NOZ and GBC-SD cells were cultured at a concentration of 1 × 10^3^ cells/well in 100 μl in 96-well culture plates and incubated with various concentrations (0, 40, 60, 80, and 100 μM) of isorhamnetin for 24, 48, and 72 h. We controlled the final DMSO level in the growth medium below 0.1%. CCK-8 (10 μl) (Yeasen, Shanghai, China, Cat number: 40203ES88) was added into each well, and the cells were incubated at 37°C for 2 h. A microplate reader (Bio-TEK, Saxony, United States) was then used to determine the absorbance at 450 nm.

### Colony Formation Assay

The NOZ and GBC-SD cells were cultured at 1,000 cells/well in 6-well culture plates and exposed to DMSO and isorhamnetin (40, 60, and 80 μM) for 10 days. Four percent paraformaldehyde was used to fix the cells for 15 min, and then staining was achieved using a 0.5% crystal violet solution for 15 min. The colony numbers (>100 cells/colony) were enumerated manually.

### Transwell Assays for Cell Migration and Invasion

We used 8.0-μm pore transwell plates (Corning, United States, Cat number: 3422) to assess cell migration, and invasion analysis was carried out in a Matrigel^®^ invasion chamber (BD Biosciences, United States, Cat number: 354480) in a 24-well plate. GBC-SD and NOZ cells were starved with serum-free medium for 24 h. We harvested the cells and seeded 2 × 10^4^ cells in serum-free medium into the upper chamber, whereas we had added 500 μl of complete medium to the lower chamber previously. In addition, the same concentrations of isorhamnetin (0, 40, 60, and 80 μM) were added to both the upper and lower chambers. Following incubation for 18 h at 37°C, cells on the upper chamber were swabbed off and the fixation of the cells on the lower surface with 4% paraformaldehyde was conducted for 15 min. Thereafter, 0.1% crystal violet staining was carried out for 15 min. Finally, the invasive or migrated cell numbers were calculated in five random fields for each group.

### Apoptosis Assay

Flow cytometry was conducted using the Annexin V-FITC/PI apoptosis detection kit (BD, United States, Cat number: 556547) according to the manufacturer’s instructions to assess cell apoptosis. We harvested the cells and rinsed them twice using ice-cold PBS, then re-suspended them in 500 μl of 1 × binding buffer after treatment with isorhamnetin (0, 40, 60, and 80 μM) for 48 h. We then stained the cells using 5 μl of Annexin V-FITC and 5 μl of propidium iodide (PI), and incubated the cells in the dark at room temperature for 15 min. The apoptosis rate was measured using flow cytometry (Cytoflex, Beckman, United States) and analyzed using the CytExpert Software (RRID:SCR_017217).

### Hoechst 33342 Staining

GBC-SD and NOZ cells were exposed to isorhamnetin (0, 40, 60, and 80 μM) for 48 h and rinsed in PBS. We fixed the cells using 4% paraformaldehyde for 15 min. Staining of the cells was performed using Hoechst 33342 (Beyotime, China, Cat number: C1029) at room temperature for 10 min. The cell fluorescence microscope (Leica Microsystems, Germany, RRID:SCR_008960) was then employed to examine the cells.

### Cell Cycle Analysis

Flow cytometry was carried out using a cell cycle and apoptosis analysis kit (Beyotime, China, Cat number: C1052) to explore the cell cycle. We treated the NOZ and GBC-SD cells with isorhamnetin (0, 40, 60, and 80 μM) for 48 h and then harvested the cells. We then fixed the cells using 70% cold ethanol at −20°C overnight. The cells were then washed using cold PBS and incubated with 500 μl of staining buffer, 25 μl of PI, and 10 μl of RNase A in the dark at 37°C for 30 min. Finally, the cell cycle distribution was evaluated using flow cytometry.

### Western Blot Assay

The GBC-SD and NOZ cells were treated with isorhamnetin (0, 40, 60, and 80 μM) for 48 h, and we harvested the cells. Lysing of these cells was conducted using RIPA buffer (Beyotime, China, Cat number: P0013C) with 1% PMSF and 1% cocktail, the BCA kit (Beyotime, Cat number: P0010S) was then employed to assess the protein concentrations. After that, 10% SDS-PAGE was used to resolve equivalent quantities of proteins that were then transferred to PVDF membranes (Millipore, United States, Cat number: IPVH00010). The membranes were then blocked using 5% skimmed milk at room temperature for 1 h. Thereafter, the membranes were incubated with the primary antibodies cleaved PARP (Cell Signaling Technology Cat# 5625, RRID:AB_10699459), BCL2 (Abcam Cat# ab32124, RRID:AB_725644), BAX (ABclonal Cat# A18642, RRID:AB_2862380), Slug (Cell Signaling Technology Cat# 9585, RRID:AB_2239535), cleaved caspases 3 (Cell Signaling Technology Cat# 9664, RRID:AB_2070042), CDK1 (Abways Cat# CY5304), cleaved caspases 9 (Cell Signaling Technology Cat# 7237, RRID:AB_10895832), cyclin B1 (ABclonal Cat# A2056), p27 (Cell Signaling Technology Cat# 3686, RRID:AB_2077850), MMP-2 (Cell Signaling Technology Cat# 13132, RRID:AB_2798128), MMP-9 (Cell Signaling Technology Cat# 13667, RRID:AB_2798289), N-cadherin (ABclonal Cat# A0433, RRID:AB_2757189), TWIST1 (ABclonal Cat# A3237, RRID:AB_2765003), P53 (ABclonal Cat# AP0263, RRID:AB_2771253), phosphorylated (p)-AKT1 (ABclonal Cat# AP0980), p-PI3KP85α/γ/β-Y467/Y199/Y464 (ABclonal Cat# AP0854, RRID:AB_2771416), E-cadherin (Cell Signaling Technology Cat# 3195, RRID:AB_2291471), PARP (Cell Signaling Technology Cat# 3195, RRID:AB_2291471), PIK3CA (ABclonal Cat# A12484, RRID:AB_2759327), AKT (Cell Signaling Technology Cat# 4691, RRID:AB_915783), caspases 9 (ABclonal Cat# A11910, RRID:AB_2758854), caspases 3 (ABclonal Cat# A2156, RRID:AB_2862975), and GAPDH (Abways Technology Cat# AB0037) at 4°C overnight. Incubation with HRP-labelled secondary antibodies was performed for 1 h. Finally, an enhanced chemiluminescence (ECL) system was used to visualize the protein bands.

### *In vivo* Tumor Xenograft Study

Four-week-old BALB/c nude female mice were acquired from the Shanghai SLAC Laboratory Animal Co., Ltd. (Shanghai, China), and the Laboratory Animal Ethical and Welfare Committee Xin Hua Hospital Affiliated To Shanghai Jiao Tong University School Of Medicine approved all the animal procedures. We subcutaneously administered 1 × 10^6^ NOZ cells resuspended in 0.2 ml PBS to nude mice on the right flank. The mice were randomly grouped into three groups (*n* = 5) after tumor formation within 3 days. The control group received an intraperitoneal injection with vehicle (1% DMSO and 99% corn oil) and the others with isorhamnetin (1 or 5 mg/kg) daily for 14 days, and body weight was measured every 2 days. We sacrificed the mice using CO2, removed the tumors, weighed them, and ground them down to extract proteins. The tumor volume (V) = (length x width^2^)/2.

### Immunohistochemistry Staining

Paraffin sections were dewaxed in water with graded dimethylbenzene and ethanol. The tissue section was incubated with citric acid (pH6.0) antigen repair solution in a microwave oven and then with 3% H_2_O_2_ solution at room temperature for 25 min. We used 3% BSA and serum to block the tissue at room temperature for 30 min. We then incubated the sections with primary antibodies Ki-67 and p-AKT1 at 4°C overnight. After that, the samples were incubated with secondary antibodies (HRP labeled) of the corresponding species at room temperature for 50 min. The segments were then stained using DAB and hematoxylin, and the sections were imaged under a microscope.

### Statistical Analysis

All assays were conducted in triplicate, and data were indicated as means ± SD. Differences between the two groups were evaluated using the Student's t-test or one-way ANOVA. All data analyses were carried out in the GraphPad Prism (RRID:SCR_002798). *p < 0.05, **p < 0.01, ***p < 0.001, and ****p < 0.0001 indicated statistical significance.

## Results

### Isorhamnetin Inhibits Proliferation of GBC Cells *in vitro*

DMSO and different isorhamnetin concentrations (0, 40, 60, 80, and 100 μM) were used for NOZ and GBC-SD treatment for 24, 48, and 72 h to detect the suppression effect of isorhamnetin on GBC cell growth. The CCK-8 assay results showed a substantial decrease in the cell viability of NOZ and GBC-SD in a time- and dose-dependent manner (Figure 1B). The IC50 values of isorhamnetin in NOZ and GBC-SD were 162.5 μM and 147.1 μM for 24 h, 103.8 μM and 87.27 μM for 48 h, and 81.2 μM and 47.52 μM for 72 h. In further experiments, isorhamnetin concentrations of 40, 60, and 80 μM, and a treatment period of 48 h were chosen to treat GBC cells based on the IC50 value. Colony formation analysis was also conducted to study the inhibiting effect of isorhamnetin on GBC cell proliferation from a long-term perspective. The data revealed that the number of colonies in NOZ and GBC-SD cells significantly decreased in a dose-dependent approach (Figures 1C,D). Collectively, these data demonstrate that isorhamnetin can exert significantly anti-tumor effects in GBC cell proliferation.

### Isorhamnetin Suppresses Migration and Invasion of GBC Cells

**FIGURE 2:**
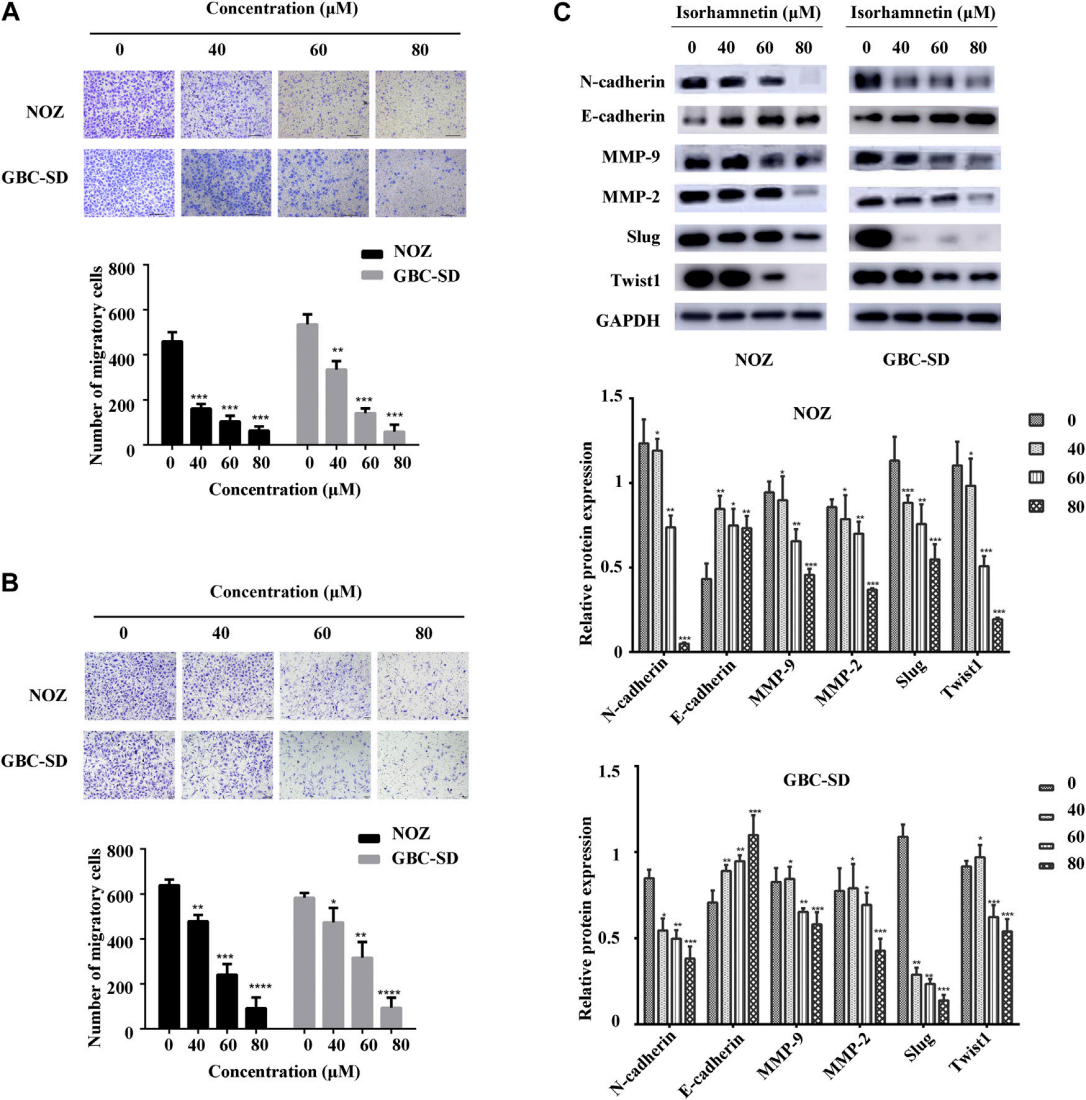
Isorhamnetin repressed GBC cell migration and invasion. **(A,B)** Cell migration and invasion of NOZ and GBC-SD detected after isorhamnetin treatment (0, 40, 60, 80 μM) for 18 h. The migrated cell numbers were computed in five random fields in each group. **(C)** The expression levels and statistic results of MMP-2, Slug, MMP-9, E-cadherin, N-cadherin, and TWIST1 in NOZ and GBC-SD after isorhamnetin treatment. GAPDH was used as the loading control. *p < 0.05, **p < 0.01, ***p < 0.001, relative to the control group.

A transwell assay for cell migration and invasion was conducted to assess the effects of isorhamnetin on the migration and invasion potential of GBC cells. The number of migrated GBC-SD and NOZ cells after treatment with isorhamnetin (0, 40, 60, and 80 μM) was substantially decreased in a dose-dependent manner (Figure 2A). Isorhamnetin also suppressed GBC-SD and NOZ cells’ invasive ability in a dose-dependent approach (Figure 2B). We then performed a western blotting assay to evaluate the expression level of proteins involved in migration and invasion after treatment with isorhamnetin. The data results showed a dose-dependent decrease in the expression levels of MMP-2, N-cadherin, MMP-9, TWIST1, and Slug in GBC-SD and NOZ cells exposed to isorhamnetin, while the expression level of E-cadherin was significantly increased (Figure 2C). These data show that isorhamnetin can suppress the metastasis of GBC cells *in vitro*.

### Isorhamnetin Induces Apoptosis in GBC Cells

**FIGURE 3:**
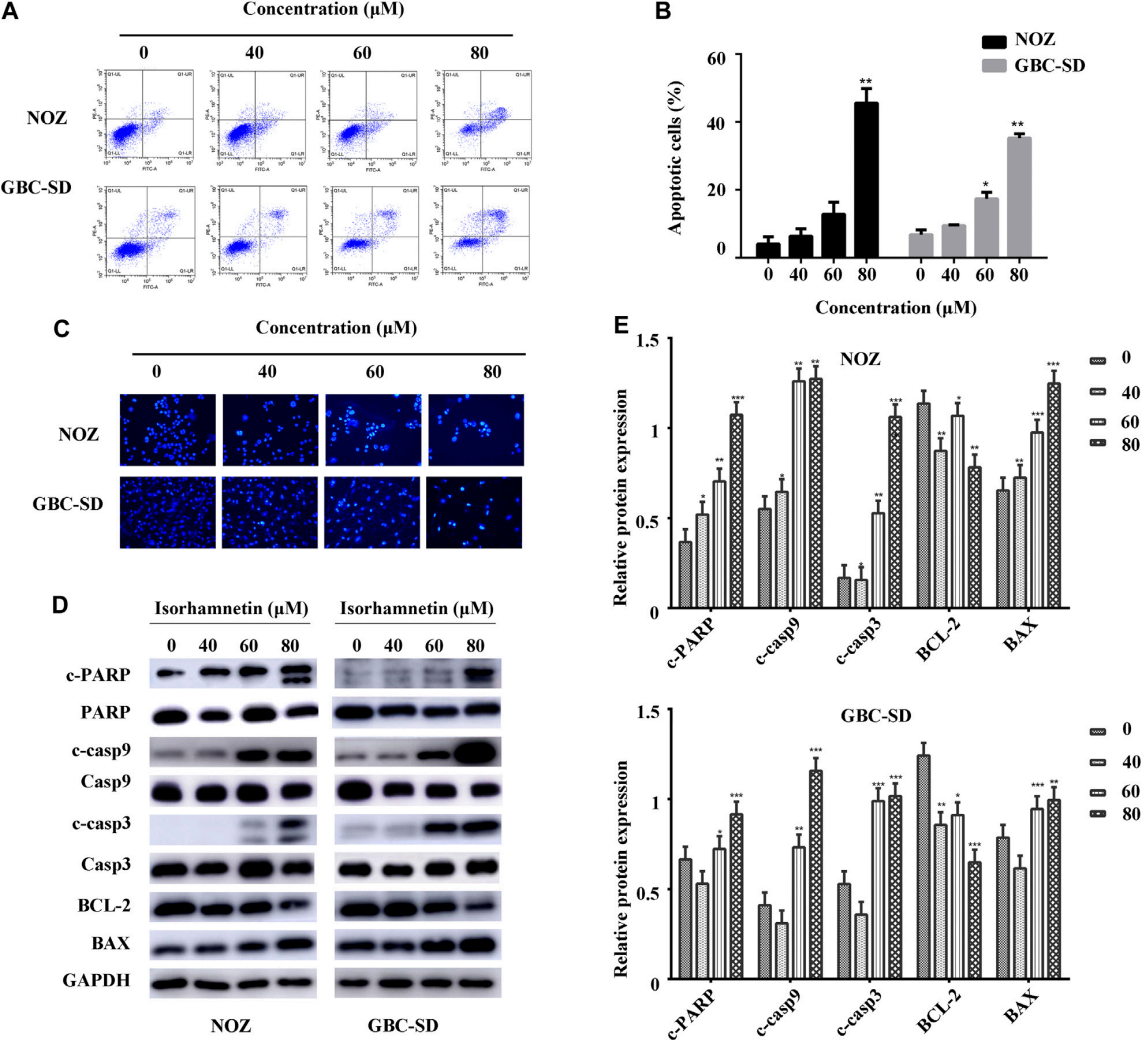
Isorhamnetin induces GBC cell apoptosis. **(A)** The stained NOZ and GBC-SD cells exposed to isorhamnetin (0, 40, 60, and 80 μM). **(B)** Apoptotic cell proportions are expressed as the mean ± SD. **(C)** The apoptotic nuclear morphology alterations were assessed using Hoechst 33342 staining via a fluorescence microscope. **(D)** The expression and **(E)** statistic results of cleaved PARP, PARP, BAX, cleaved caspases 9, caspases 9, BCL-2, cleaved caspases 3, and caspases 3 in NOZ and GBC-SD after isorhamnetin treatment. *p < 0.05, **p < 0.01 relative to the control group.

We conducted Annexin V-FITC/PI staining and flow cytometry to examine the apoptosis change in GBC cells treated with isorhamnetin (0, 40, 60, and 80 μM) for 48 h. The living cells in the lower left quadrant significantly reduced after treatment with isorhamnetin. In contrast, the early and late apoptotic cells in the lower right and upper right quadrant substantially increased in a dose-dependent approach (Figures 3A,B).

Hoechst 33342 staining was conducted to further identify cell apoptosis from a morphological perspective. The control NOZ and GBC-SD cells exhibited a round and homogeneous distribution of chromatin in the nucleus, while chromatin condensation and nuclear fragmentation were reported in isorhamnetin-treated cells (40, 60, and 80 μM) in a dose-dependent approach (Figure 3C).

A western blot assay was performed to investigate apoptosis-associated protein expressions in GBC-SD and NOZ cells following treatment with isorhamnetin (0, 40, 60, and 80 μM). The results demonstrated that the expression level of cleaved PARP (c-PARP), BAX, cleaved caspases 9, and cleaved caspases 3 substantially increased, while the expression level of BCL-2 significantly decreased (Figures 3D,E). There was no substantial change in total PARP, caspases 9, and caspases 3. Taken together, these findings suggest that isorhamnetin could induce the mitochondrial-dependent apoptosis of GBC cells in a dose-dependent approach *in vitro*.

### Isorhamnetin Triggers Cell Cycle Arrest in the G2/M Phase

**FIGURE 4:**
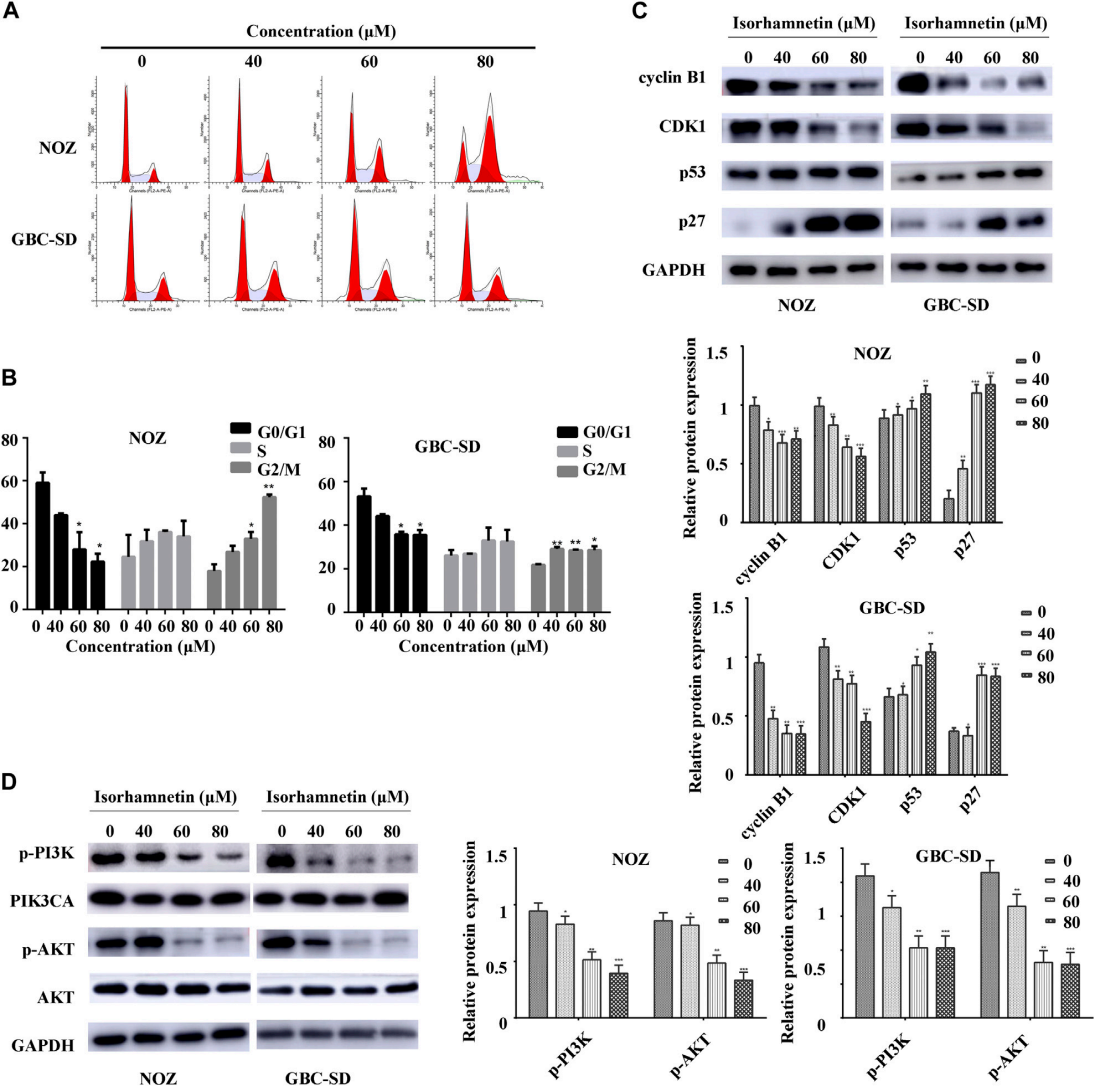
Isorhamnetin triggers cell cycle arrest in the G2/M phase and regulates PI3K/AKT cascade. **(A)** Cell cycle phases of NOZ and GBC-SD cells exposed to isorhamnetin (0, 40, 60, and 80 μM) for 48 h analyzed using flow cytometry. **(B)** Cell cycle phase distribution indicated as the mean ± SD (*n* = 3). **(C)** Levels of expression of cyclin B1, CDK1, p27, and p53 were determined using western blot assessment. **(D)** The expression levels of p-PI3KP85α/γ/β-Y467/Y199/Y464, p-AKT1 (T450), PIK3CA, and AKT were detected. *p < 0.05, **p < 0.01 relative to the control group.

A flow cytometry assay was conducted to investigate cell cycle distribution. The findings revealed that after isorhamnetin exposure (40, 60, and 80 μM), the proportion of NOZ and GBC-SD cells in the G2/M phase was significantly elevated in a dose-dependent approach (Figure 4A), while the percentage of NOZ and GBC-SD cells in the G0/G1 phase was substantially decreased (Figure 4B). A western blotting assay was conducted to determine the expression level of cell cycle-associated proteins to examine the potential molecular mechanism. As shown in Figure 4C, isorhamnetin significantly suppressed the expression of CDK1 and cyclin B1 levels in a dose-dependent approach, whereas the expression of CDK inhibitor p27 and p53 was upregulated. Therefore, these results reveal that isorhamnetin can inhibit GBC cell proliferation via G2/M cell cycle arrest.

### Anti-Tumor Effect of Isorhamnetin via PI3K/AKT Pathway

**FIGURE 5:**
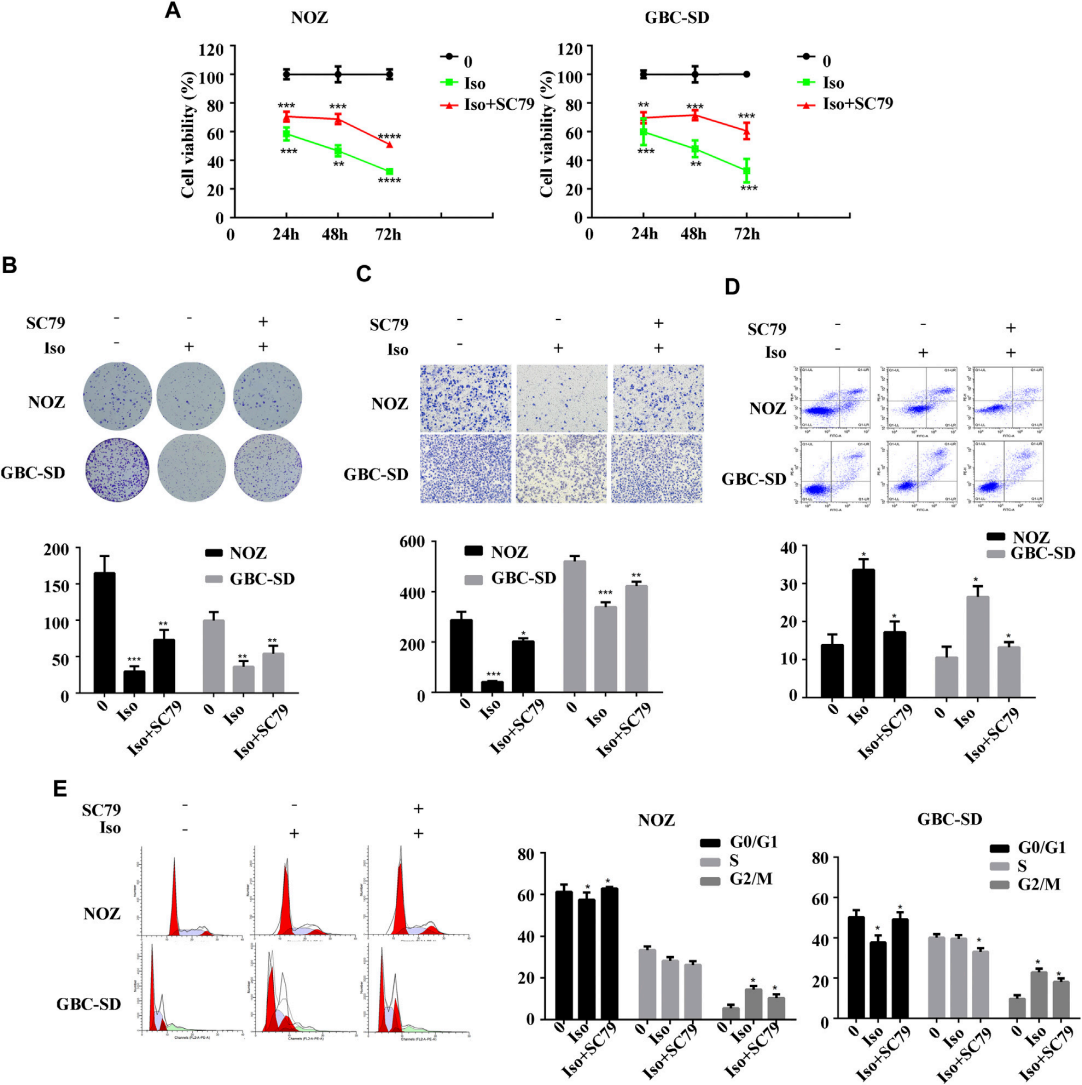
Isorhamnetin inhibits GBC cell proliferation and metastasis via the PI3K/AKT pathway. **(A)** The cell viability of GBC cells assessed using CCK-8 assay after isorhamnetin treatment (0 and 80 μM) with or without SC79 pretreatment for 1 h. **(B)** Colony formation assay of NOZ and GBC-SD cells for 10 days. **(C)** Cell migration, **(D)** apoptosis, and **(E)** cell cycle were detected. *p < 0.05, **p < 0.01, ***p < 0.001 relative to the control group.

**FIGURE 6:**
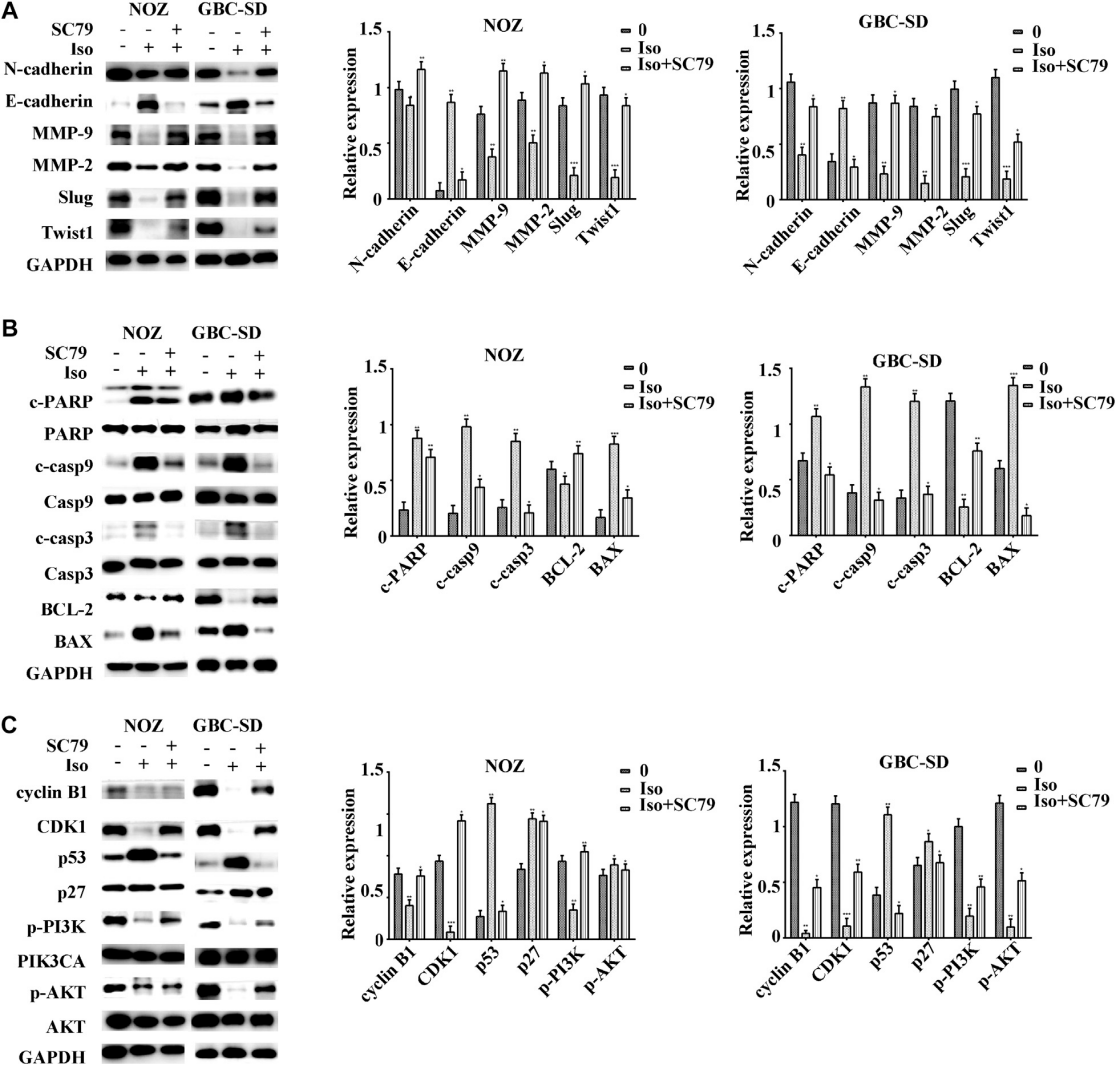
Isorhamnetin inhibits GBC cell proliferation and metastasis via the PI3K/AKT pathway. After pretreatment with SC79 for 1 h, the expression levels of **(A)** MMP-2, Slug, MMP-9, E-cadherin, N-cadherin, TWIST1, **(B)** cleaved PARP, PARP, BAX, cleaved caspases 9, caspases 9, BCL-2, cleaved caspases 3, caspases 3, **(C)** cyclin B1, CDK1, p27, p53, p-PI3K, p-AKT1, PIK3CA, and AKT were detected and shown with statistic results. *p < 0.05, **p < 0.01, ***p < 0.001 relative to the control group.

The PI3K/AKT signaling cascade was examined using a western blot assay to explore the molecular mechanism of isorhamnetin-mediated repression of proliferation and metastasis. The expression level of p-PI3KP85α/γ/β-Y467/Y199/Y464, p-AKT1 (T450) significantly decreased in a dose-dependent approach in NOZ and GBC-SD cells treated with isorhamnetin (0, 40, 60, and 80 μM) for 48 h (Figure 4D). SC79, an AKT agonist, was used to perform a rescue experiment. The effects of isorhamnetin (80 μM) on cell proliferation, apoptosis, G2/M cell cycle arrest (Figures 5A–E), and the PI3K/AKT pathway (Figures 6A–C) in GBC cells were eliminated after pretreatment with SC79 for 1 h. Overall, these data imply that the PI3K/AKT signaling cascade plays an essential role in the isorhamnetin-mediated anti-tumor effect in GBC cells.

### Isorhamnetin Inhibits Tumor Growth *in vivo*

**FIGURE 7:**
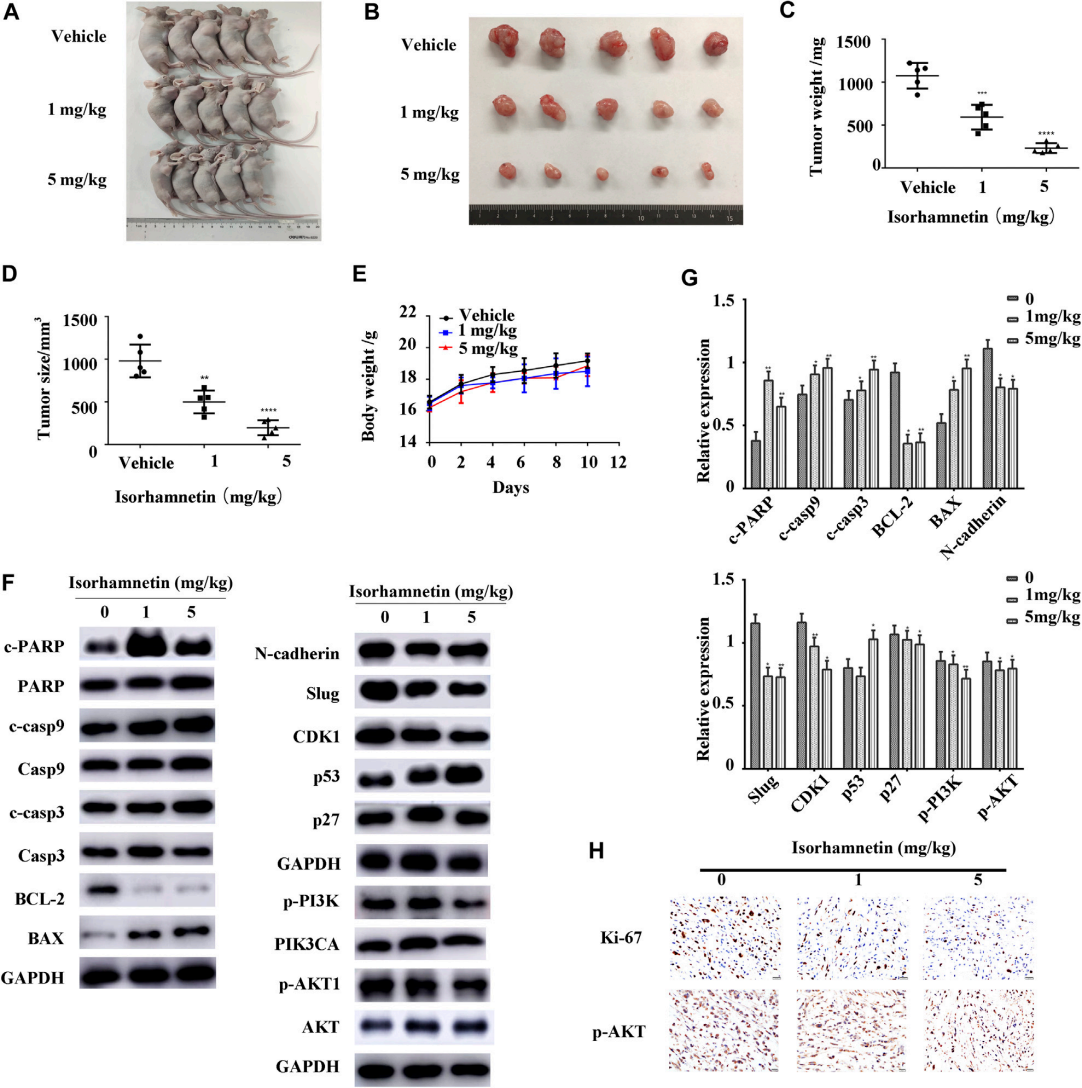
Isorhamnetin inhibits tumor growth *in vivo*. **(A,B)** We subcutaneously administered NOZ cells into the right flank of nude mice. We then intraperitoneally administered 0.2 ml of vehicle (1% dimethyl sulfoxide and 99% corn oil) or isorhamnetin (1, 5 mg/kg) to the mice every 2 days for 14 days. Necropsy images of mice and tumors were then presented. **(C,D)** Tumor weight and size measured after tumor harvesting. ***p < 0.001 relative to the control group. **(E)** The body weight of mice was monitored during the experiment period. **(F,G)** Expression levels of p-PI3KP85α/γ/β, PIK3CA, cleaved PARP, PARP, cleaved caspases 9, caspases 9, p27, cleaved caspases 3, caspases 3, p53, BAX, BCL-2, N-cadherin, Slug, CDK1, p-AKT1, and AKT were detected. **(H)** IHC staining of Ki-67 and p-AKT1 expressions in subcutaneous xenograft tumors (magnification = 400 x) **p < 0.01, ***p < 0.001, ****p < 0.0001 relative to the control group.

A xenograft mouse model was developed to elucidate the isorhamnetin-mediated anti-tumor effects in GBC *in vivo*. The tumor size and weight significantly reduced in a dose-dependent approach after 14 days of isorhamnetin injection (DMSO, 1 mg/kg, 5 mg/kg) (Figures 7A–D). Besides, there was no apparent weight loss or mortality in mice undergoing isorhamnetin treatment, suggesting that isorhamnetin is safe for *in vivo* use with no unnecessary side effects (Figure 7E). Furthermore, the western blot assay results indicate that isorhamnetin treatment substantially elevated the expression of cleaved PARP, p53, cleaved caspases 9, BAX, cleaved caspases 3, and p27, but downregulated BCL-2, N-cadherin, Slug, CDK1, p-PI3KP85α/γ/β, and p-AKT1 in dissected tumor tissues (Figures 7F,G). As shown in Figure 7H, IHC tumor sections staining had a reduced expression level of proliferation marker Ki-67 and p-AKT1, because of isorhamnetin treatment. Taken together, these results demonstrate that isorhamnetin may inhibit GBC growth *in vivo* and induce apoptosis via the PI3K/AKT signaling cascade.

## Discussion

Recently, isorhamnetin has been reported to exert inhibitory effects on various tumors. However, the effects of isorhamnetin on GBC cells have not been widely studied. This study elucidated how isorhamnetin stimulates apoptosis and G2/M phase arrest, and inhibits metastasis of human GBC cells *in vitro* and *in vivo* in a dose-dependent approach.

The anti-proliferation properties of isorhamnetin were elevated as shown by CCK8 and colony formation assays. Isorhamnetin has been identified to effectively suppress GBC-SD and NOZ cell proliferation in a time- and dose-dependent manner. Using a transwell assay, we also found that isorhamnetin can significantly inhibit the migration and invasion capability of NOZ and GBC-SD. Epithelial-mesenchymal transformation (EMT) plays an essential role in the migration and invasion of cancer cells (Zheng et al., 2015). Matrix metalloproteinase (MMPs) including MMP2 and MMP9, N-cadherin, and transcription factors TWIST and Slug are the major components associated with cell metastasis and are considered to be epithelial cell biomarkers (Xie et al., 2019). A western blot assay demonstrated that isorhamnetin significantly suppresses the expression of Slug, N-cadherin, MMP2, MMP9, and TWIST1 while it upregulated E-cadherin.

A flow cytometric assay and Hoechst 33342 staining were performed to confirm if isorhamnetin inhibits NOZ and GBC-SD by inducing apoptosis. We found that isorhamnetin stimulated apoptosis in GBC cells in a dose-dependent approach. The intrinsic mitochondrial axis and the extrinsic death receptor-induced pathway are the two main cascades that cause apoptosis (Call et al., 2008). Intracellular toxicity stimuli initiate intrinsic apoptosis. Caspase 9 is activated to trigger the downstream proteins such as caspases 3 and PARP (Nuñez et al., 1998). Moreover, the BCL-2 family is an essential regulator of the intrinsic apoptotic pathway, including pro-survival protein BCL-2 and pro-apoptotic protein BAX (Czabotar et al., 2014). In this study, we revealed that isorhamnetin substantially decreases the BCL-2 expression and elevates the expression of cleaved PARP, caspases 3 and 9, and BAX in NOZ and GBC-SD, suggesting that isorhamnetin can induce GBC cells apoptosis via the intrinsic mitochondrial pathway.

The cell cycle distribution was investigated to determine the potential mechanism of the anti-proliferation effects of isorhamnetin. In this study, isorhamnetin triggered the G2/M phase arrest of NOZ and GBC-SD cells. During mitosis, cyclin-dependent kinase 1 (CDK1) plays a vital role in the transition from the G2 to M phase, interacting with cyclin B1 to form a heterodimer (Castedo et al., 2002). After DNA damage, p53 is activated via negative regulation of CDK1 and cyclin B1 to trigger G2/M phase arrest (Agarwal et al., 1998). CDK inhibitor p27 also plays an important role in inhibiting CDK1 activity at G2/M (Nakayama et al., 2004). In this study, the western blotting assay demonstrated that after isorhamnetin treatment in NOZ and GBC-SD cells, the level of expression of CDK1 and cyclin B1 reduced while that of p53 and p27 increased. These findings demonstrate that isorhamnetin can trigger G2/M phase arrest of GBC via p53-mediated downregulation of CDK1 and cyclin B1.

Isorhamnetin is known to induce essential anti-tumor effects via various signaling cascades, including the MEK, PI3K/AKT, and NF-κB signaling cascades. The PI3K/AKT signaling cascade modulates cell growth, differentiation, and frequent change in various human cancers (Fresno Vara et al., 2004). It has been reported that p53 degradation is induced by the interaction of AKT and DNA damage (Pan et al., 2017). In the present study, western blot data demonstrated that isorhamnetin upregulated the expression of p53 and downregulated p-PI3K and p -AKT1 in NOZ and GBC-SD. The rescue experiments indicated that an AKT activator SC79 can significantly reverse isorhamnetin’s anti-tumor effects on GBC cells by inhibiting proliferation and metastasis, inducing apoptosis and G2/M cycle arrest, and downregulating PI3K/AKT signaling pathways *in vitro*. Taken together, these results demonstrate that isorhamnetin regulates apoptosis, cell cycle, and metastasis of GBC by downregulating PI3K/AKT signaling pathways.

The xenograft model was established to elucidate the anti-tumor effects of isorhamnetin *in vivo*. In this study, we confirmed that isorhamnetin inhibits tumor growth in a dose-dependent approach. Moreover, there was no substantial weight loss observed in the three groups, indicating safety and the absence of side effects *in vivo*. We performed a western blot assay to confirm if isorhamnetin induces inhibitory effects via the same mechanism as seen *in vitro*. Representative proteins of PI3K/AKT pathways were selected, and the results showed that expressions of BAX, cleaved PARP, cleaved caspases 9, p53, and cleaved caspases 3 were upregulated while those of BCL2, N-cadherin, Slug, CDK1, cyclin B1, p-PI3K, and p-AKT1 were downregulated. These findings imply that isorhamnetin suppresses GBC tumor growth *in vivo* via downregulating PI3K/AKT signaling cascades. Though we have used multiple techniques to validate our findings *in vivo* and *in vitro*, there are still many limitations. The side effects of isorhamnetin need to be studied extensively, histochemical staining of the heart, brain, and lung of nude mice are essential. We will explore the deeper mechanism of isorhamnetin’s anti-tumor effects, and study the pathway in GBC patient tissue samples in the future.

In conclusion, our study verifies the biological functions and clinical value of isorhamnetin in GBC development for the first time. We demonstrate that isorhamnetin may suppress proliferation and metastasis, and trigger G2/M phase arrest of GBC cells by downregulating the PI3K/AKT signaling cascade without any side effects, which could provide a novel potential treatment strategy for GBC patients.

## Data Availability

The original contributions presented in the study are included in the article/Supplementary Material, further inquiries can be directed to the corresponding authors.
