# Corrigendum: Isorhamnetin Inhibits Human Gallbladder Cancer Cell Proliferation and Metastasis *via* PI3K/AKT Signaling Pathway Inactivation

**DOI:** 10.3389/fphar.2021.792330

**Published:** 2021-10-20

**Authors:** Tianyu Zhai, Xiaoyu Zhang, Zhenyu Hei, Longyang Jin, Chao Han, Audrey Tsznam Ko, Xiaofeng Yu, Jiandong Wang

**Affiliations:** ^1^ Department of General Surgery, Xinhua Hospital, Affiliated to Shanghai Jiao Tong University School of Medicine, Shanghai, China; ^2^ Shanghai Research Center of Biliary Tract Disease, Shanghai, China; ^3^ Shanghai Key Laboratory of Biliary Tract Disease Research, Shanghai, China; ^4^ Department of Colorectal Surgery, The Sixth Affiliated Hospital of Sun Yat-sen University, Guangzhou, China; ^5^ Department of General Surgery, Shanghai General Hospital, Shanghai Jiao Tong University School of Medicine, Shanghai, China; ^6^ Faculty of Medicine, Imperial College London, London, United Kingdom; ^7^ Department of General Surgery, People’s Hospital of Gaoxin District, Suzhou, China

**Keywords:** isorhamnetin, gallbladder cancer, tumor progression, PI3K/Akt pathway, apoptosis

In the published article, there was an error in the affiliations order. Affiliations 1 and 3 were interchanged, and the superscript numbers 1 and 3 in the authors’ affiliations list were updated accordingly.

The authors apologize for this error and state that this does not change the scientific conclusions of the article in any way. The original article has been updated.

